# Stable-Isotope Probing of Human and Animal Microbiome Function

**DOI:** 10.1016/j.tim.2018.06.004

**Published:** 2018-12

**Authors:** David Berry, Alexander Loy

**Affiliations:** 1 Division of Microbial Ecology, Department of Microbiology and Ecosystem Science, Research Network Chemistry Meets Microbiology, University of Vienna, Althanstrasse 14, Vienna, Austria

**Keywords:** gut microbiota, single-cell imaging, RNA-SIP, NanoSIMS, Raman microspectroscopy

## Abstract

Humans and animals host diverse communities of microorganisms important to their physiology and health. Despite extensive sequencing-based characterization of host-associated microbiomes, there remains a dramatic lack of understanding of microbial functions. Stable-isotope probing (SIP) is a powerful strategy to elucidate the ecophysiology of microorganisms in complex host-associated microbiotas. Here, we suggest that SIP methodologies should be more frequently exploited as part of a holistic functional microbiomics approach. We provide examples of how SIP has been used to study host-associated microbes *in vivo* and *ex vivo*. We highlight recent developments in SIP technologies and discuss future directions that will facilitate deeper insights into the function of human and animal microbiomes.

## Highlights

Meta-omic analysis has produced an immense amount of knowledge on the composition and development of human and animal microbiomes, yet most functions encoded in the metagenome are unknown.

Stable-isotope probing (SIP) is a powerful strategy for elucidating the ecophysiology of individual microorganisms in complex host-associated microbiotas.

An array of SIP approaches has been developed that allow testing of hypotheses from meta-omic datasets and discovery of new functions.

SIP is being used to determine the nutrient-utilization strategies of microbes *in vivo* and *ex vivo* as well as ecological relationships such as competition, cooperation, and cross-feeding.

## Understanding Ecophysiological Mechanisms of Microbiota Functionality

Humans and other animals live in an intimate relationship with their microbiota – a community of trillions of single-celled organisms, most of which reside in the intestinal tract. This symbiosis is characterized by bidirectional interactions between host and microbe. Genetics, lifestyle, and health of the host (e.g., diet, living environment, social behavior, infection, medication) determine the presence and dynamics of ecological niches in different body habitats that microorganisms can occupy for growth. Besides their important role in shaping and modulating the immune system, it is through their metabolic functions that symbiotic microorganisms impact the physiology and health of their host. Microorganisms have, or can rapidly acquire or evolve, the necessary enzymes for metabolizing virtually any substrate – be it a dietary, a host-derived or a xenobiotic compound [1]. They remove, create new, and modify metabolites and thereby shape the chemical landscape of their habitat by altering nutrient composition, bioactive compounds, and signaling molecules for other microorganisms and their host [2].

**Figure 1:**
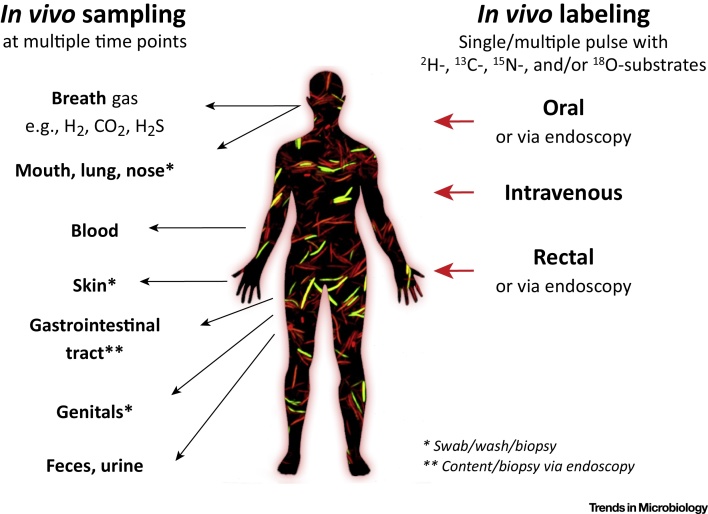
*In Vivo* Stable-Isotope Probing (SIP). Options for mode of administration of stable-isotope-labeled substrate as well as sampling for molecular and chemical analysis.

Recent developments in DNA/RNA sequencing and bioinformatics now allow for strain-resolved metagenomic and metatranscriptomic analyses 3, 4, 5. This promises unprecedented insights into the diversity and evolution of the microbiome, including differences between individuals and social groups, lifetime dynamics, acquisition-loss-dispersal events, and associations between individual strains with specific host phenotypes or diseases [6]. However, our interpretation of microbial metabolism from sequencing data alone remains remarkably limited because the functions of most microbial genes are unknown 7, 8. Only between 30 and 60% of the genes in the expanding human microbiome gene catalog can be assigned to known orthologous groups 7, 9, and only a minute proportion of these genes has been characterized biochemically 10, 11. Even the population of *Escherichia coli* strains, the best-studied microbial model species, still contains thousands of genes with unknown functions [12]. Only a small fraction of an individual microorganism’s genetic repertoire is transcribed in a fecal sample [13]. This dramatic lack of understanding of microbial functions calls for integration of available technologies such as omics approaches, use of defined experimental systems (e.g., *in vitro* gut bioreactors, gnotobiotic animals), and human intervention studies 7, 8. Here, we suggest that stable-isotope probing (SIP) methodologies should be more frequently exploited in such a holistic, functional microbiomics approach [14]. Stable isotopes have been used for decades in applied human nutrition [15], yet still rarely in microbiome research 15, 16. Specific physiological capabilities of microbiota members can be studied *in situ* by SIP without prior knowledge of the biochemical and genetic basis of the involved enzymatic machinery [17]. Additionally, the metabolome contains many uncharacterized metabolites, and SIP also offers an opportunity to trace active metabolic production even without knowing the identity of the produced metabolites. SIP involves the administration of an isotopically labelled substrate, followed by tracing the fate of the isotope label through the host’s body and the microbiota. Microorganisms that have incorporated the isotope label in their biomass are identified by molecular biology methods or by single-cell imaging. The physiology under investigation is determined by substrate type, isotope label (^18^O, ^13^C, ^15^N, ^2^H), dosage, and mode of application (ingestion or endoscopy; orally, rectally, intravenously), as well as the physiological and health status of the host (Figure 1).

## The Holistic Concept of Isotope Probing

**Figure 2:**
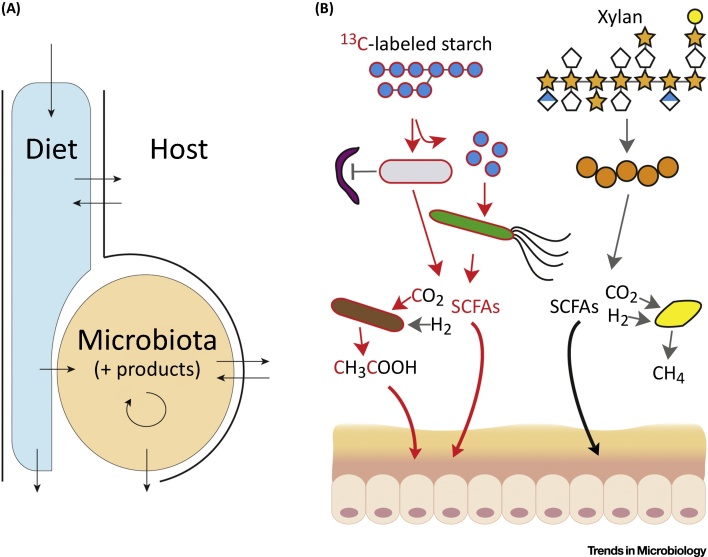
Mass Flows in the Host-Associated Microbiota. (A) Schematic of general mass flows in the gut ecosystem, from diet to and between host and microbiota. (B) Conceptual example of how stable-isotope probing (SIP) can be used to specifically track isotopically labeled microbes involved in ^13^C-starch degradation or downstream in the trophic cascade, and to follow the fate of starch degradation metabolism and products in the microbiota and host (red indicates ^13^C in compounds and biomass). Intestinal metabolites are absorbed and metabolized by the epithelium and/or further distributed to peripheral host tissues and organs (e.g., liver, kidney, brain) by the circulation. SCFA, short-chain fatty acid.

The microbiota is typically thought of in taxonomic and genetic terms – that is, its phylogenetic/taxonomic composition, or the genetic content of the microbiome – and how it changes under different conditions, such as with diet shift or in different disease states. However, the microbiota–host symbiosis can also be conceptualized as a mass balance of elements (Figure 2). There are flows of macroelements, such as C, H, O, N, S, and P, as well as microelements, such as Fe, Zn, and Mg, into and out of the many cells of the microbiota as well as between the microbial ecosystem and its host, analogous to global biogeochemical cycles such as the carbon or nitrogen cycles. These elements provide the basis for molecules that have important structural, metabolic, and immunogenic properties. Both microorganisms and macroorganisms are generally constrained homeostatically by a certain ratio, or stoichiometry, of these elements, and therefore an elemental view of the symbiosis can be very powerful in thinking about growth limitations and system efficiency and function. A recent example of considering the importance of thinking about mass balances is the discovery that microbial load (i.e., overall microbial mass) is a determinant of enterotype status, a concept that had been established by analysis of the relative taxonomic composition of the gut microbiota [18]. Taking account of mass and mass balances will be an important step to better understand the symbiosis between the microbiota and host. Stable isotopes are a powerful tool to quantify these mass balances and fluxes (techniques to do so are reviewed in [14]).

Diet is the source of new elements that are introduced into the host–microbiome system. In the upper gastrointestinal (GI) tract, the host secretes digestive enzymes to break down and absorb dietary components. As the rest of the dietary intake moves down the GI tract, the intestinal microbiota catalyzes the degradation of dietary compounds resistant to digestion by the host – such as cellulose, hemicellulose, xylan, and pectin [19]. The microbiota itself is likely a major sink for dietary macroelements and microelements as it needs to continuously renew itself. Dying microbial cells and exopolysaccharides, such as those produced by bifidobacteria and lactobacilli, can provide a source of nutrients for growing cells [20], although it is not yet known how extensive the turnover of microbial biomass and nutrient recycling is *in vivo*. There are also mass flows from the host to the microbiota in the form of secreted compounds via mucus such as mucin glycoproteins [21], sloughed epithelial cell components such as ethanolamine [22], as well as secretions from the bile duct like primary bile acids [23].

The microbiota not only regenerates its own biomass but, from fermentation and anaerobic respiration of diet- and host-derived compounds, also produces compounds such as short-chain fatty acids (SCFAs), trimethylamine, and hydrogen sulfide that are absorbed by the host. The vast majority of metabolites in humans are uncharacterized, and it is likely that many of these are of microbial origin. These compounds can have important epigenetic and regulatory effects on the host 24, 25 and are also often important substrates for host cell metabolism [26]. SCFAs are particularly abundant microbial products in the gut. They interact with G-protein-coupled-receptors and signal to the host. Butyrate is used by colonocytes for energy generation, propionate and succinate are substrates for gluconeogenesis, and acetate is used for lipogenesis in the liver. The flux of microbial products contributes to a major portion of nutrition/energy to ruminant animals and is estimated to contribute about 10% to the human energy balance [27], though it is not known how this is altered by microbial characteristics (composition, activity, and efficiency) and diet. Additionally, the host may be able to alter the microbiota by depriving it of nutrients such as iron and zinc [28] or regulating the composition or amount of secretions that are used as nutrient sources for the microbiota [29].

The principles of ecological stoichiometry can be applied to understand and quantify processes. The use of isotopes as tracers of these flows is vital to applying this view. Isotope tracers have been used extensively in environmental research to study microbial ecophysiology and nutrient cycling [30]. Isotopes also have a long history in human physiological research, for example for the estimation of protein synthesis rates, metabolite flux, and nutrient metabolism 31, 32, 33, 34. Isotope tracer tests have been used to monitor gastrointestinal function, including noninvasive breath tests such as the ^13^C urea breath test for diagnosing *Helicobacter pylori* infections as well as tests for orocecal transit time and small intestinal malabsorption [16]. However, these studies largely ignore the microbiota or treat it as a black box. Exceptions where stoichiometry and nutrient flux have been used within studies on the microbiota are reviewed below.

## SIP Approaches to Study the Microbiota

**Figure 3:**
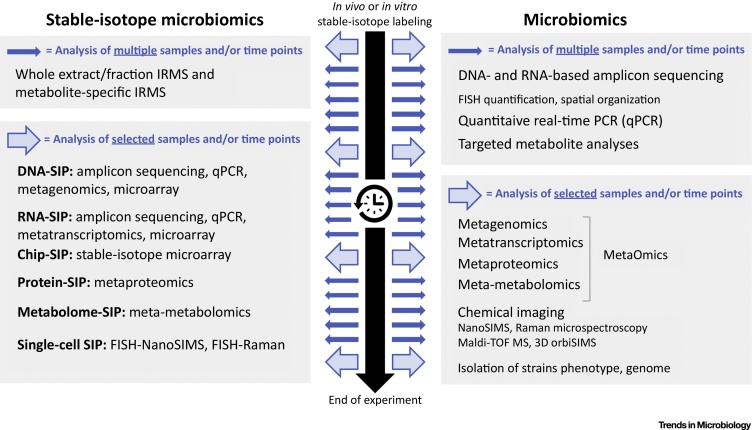
Outline of a Holistic Stable-Isotope Probing (SIP) Experiment. SIP technologies are integrated with multi-omics, microscopic and chemical imaging to provide a comprehensive picture of the microbiome dynamics and activities, and specific identities and physiological interactions of isotope-labelled microorganisms. Thick black arrow with clock indicates the timeline of an experiment. Thick and thin blue arrows indicate sampling time-points during the experiment for different analyses. IRMS, isotope ratio mass spectrometry. FISH, fluorescence *in situ* hybridization.

SIP is based on the assimilation of stable isotopes (most commonly ^13^C, ^15^N, ^18^O, and ^2^H) into newly-synthesized microbial biomass and the subsequent detection of isotopes in bulk biomass, single cells, or cellular components using specialized instrumentation. This is often combined with molecular techniques such as fluorescence *in situ* hybridization (FISH) or sequencing-based approaches such as amplicon sequencing of marker genes and metagenomics to identify taxa that have incorporated the isotope (reviewed in 15, 16, 35, 36, 37). A holistic SIP experiment would ideally combine different methodologies in a systems biology approach (Figure 3). However, SIP methods are time-consuming and often require expensive, high-end instrumentation, which severely limits the number of samples that can be analyzed and thus requires careful experimental planning. Most SIP studies have focused on microbial utilization of specific, fully isotopically labeled compounds (substrate-mediated SIP). However, it is also possible to use compounds as general activity markers such as heavy water (^2^H_2_O), ^13^CO_2_, or total ^15^N-labeling, as active cells will incorporate hydrogen from water, ^13^C-carbon during autotrophic or heterotrophic CO_2_ assimilation, or ^15^N-nitrogen from a completely ^15^N-labeled diet into newly-synthesized biomass, irrespective of their physiology 38, 39.

In DNA- and RNA-SIP, microbial populations that have assimilated stable isotopes (typically ^13^C or ^15^N) are identified by separating nucleic acids into unlabeled and labeled fractions (also termed ‘light’ and ‘heavy’ fractions) by isopycnic ultracentrifugation due to their different densities. The fractions can then be analyzed by sequencing-based approaches to reveal the phylogenetic and functional gene composition or to reconstruct the genomes of individual microbial populations from the metagenomes. Alternatively, nucleic acids can be probed by hybridizing them to specially designed DNA microarrays, and isotope composition of individual probe spots can be quantified using spatially resolved elemental analysis [40]. In phospholipid-derived fatty acid-(PLFA)-SIP, PLFAs are separated by gas chromatography, and isotopes are measured with isotope ratio mass spectrometry [41]. This method is extremely sensitive and can measure low enrichments in stable isotopes, but has a limited taxonomic resolution as many fatty acids are conserved among microbes. Protein-SIP evaluates stable isotope incorporation into newly-synthesized proteins by mass spectrometry [42]. As it is dependent on peptide identification, it is critical to have a comprehensive reference database (such as from metagenome sequencing) of the community that is being probed. Though targeted metabolite analysis is frequently performed in combination with SIP [21], there are thus far few examples of combining SIP with untargeted metabolomics [43], and this is an exciting area for future development.

More recently, SIP approaches have been developed using two single-cell technologies: Raman microspectroscopy and nano-scale resolution secondary ion mass spectrometry (NanoSIMS) 8, 44, 45 Raman microspectroscopy produces a chemical fingerprint of single cells based on the inelastic scattering of photons from molecular bonds [46]. The resulting fingerprint is a complex spectrum, but ^13^C and ^2^H incorporation can be readily discerned by shifts in characteristic peaks in the spectrum. Importantly, as the measurement is nondestructive, cells can be processed postmeasurement, allowing for subsequent sorting and molecular analysis [38]. In NanoSIMS, a high-energy primary ion beam is used to release atomic and small molecular ions from the cell, and ions are separated and measured by mass spectrometry [45]. Both of these approaches can be combined with FISH to simultaneously identify and quantify the activity of single cells in complex microbial communities. Comprehensive multi-omics analyses are being applied widely 6, 47, and many physiological hypotheses are generated from these large, complex datasets. Inclusion of SIP approaches in a study workflow will be an important step forward for testing these hypotheses (Figure 3).

## Examples of SIP of the Gut Microbiota

SIP has been successfully used to uncover the *in situ* ecophysiology of a number of clades of intestinal microorganisms using ^13^C-labeled dietary- and host-derived compounds. By administering ^13^C-labeled glucose to an *in vitro* human gut model reactor, Egert *et al.* demonstrated that *Streptococcus bovis* and *Clostridium perfringens* were the most active glucose fermenters using RNA-SIP [48]. They then used nuclear magnetic resonance spectroscopy to evaluate the fate of the carbon from the fermented glucose and were able to identify lactate, acetate, butyrate, and formate as the principal fermentation products, which together constituted over 90% of the ^13^C-carbon balance. Glucose utilization has also been studied in the murine gut microbiota using anaerobic incubations of fecal pellets with ^13^C-labeled glucose followed by RNA-SIP, with *Allobaculum* spp. being identified as active glucose utilizers, and with lactate, acetate, and propionate being the principal end-products of glucose fermentation [49]. ^13^C-labeled sialic acid, a monosaccharide found in many glycans in the body as well as in milk, was used to identify *Prevotella* spp. as the most abundant utilizers of sialic acid in the piglet cecal microbiota by *in vitro* incubation followed by RNA-SIP [50]. In an *in vivo* mouse model of total parenteral nutrition, an intravenously administered ^13^C-labeled leucine tracer was used to demonstrate that members of the Enterobacteriaceae utilize blood-derived amino acids that leak into the gut, which may contribute to their bloom during small intestinal inflammation associated with parenteral nutrition [51]. Using ^13^C-labeled bicarbonate, several acetogens in the kangaroo forestomach, such as *Blautia* spp. as well as several taxa not previously reported to be hydrogenotrophic organisms, were identified with RNA-SIP [52]. In this study, the researchers were also able to link the activity of acetogenic communities with overall methane output, suggesting competition between reductive acetogens and methanogens for hydrogen.

Though study of complex carbon utilization using stable isotopes has been limited by the availability of isotope-labeled compounds, a number of studies have been conducted using commercially available substrates as well as by alternative methodologies. Anaerobic *in vitro* fermentation of murine stool with ^13^C-labeled potato starch followed by RNA-SIP revealed that members of the Prevotellaceae and Ruminococcaceae were primary assimilators of resistant starch, and that acetate, propionate, and butyrate were among the fermentation products, as determined by high-performance liquid chromatography–isotope ratio mass spectrometry (HPLC–IRMS) analysis [53]. By administering ^13^C-labeled starch to an *in vitro* human gut model reactor, *Ruminococcus bromii* was identified with RNA-SIP to be an important primary of degrader of starch, and was accompanied by the production of acetate, butyrate, and propionate [54]. Interestingly, this analysis also indicated that other taxa, such as *Prevotella* spp., *Bifidobacterium adolescentis*, and *Eubacterium rectale*, may be involved in cross-feeding on sugars released from starch degradation or in the downstream trophic chain resulting from *R. bromii* activity. By amending a human gut model reactor with ^13^C-labeled galacto-oligosaccharides (GOSs), which are considered to be a prebiotic compound, a number of bifidobacteria as well as lactobacilli were found to be important GOS-assimilators using RNA-SIP [55]. The authors also observed metabolic cross-feeding. Addition of a ^13^C-labeled version of another prebiotic compound, inulin, to mouse diet led to the identification of *Bacteroides uniformis*, *Blautia gluceraseae*, *Clostridium indolis*, and *Bifidobacterium animalis* as major *in vivo* utilizers of inulin in the mouse cecal community using RNA-SIP [56].

As an alternative to directly adding a labeled complex compound, it is also possible to add simple precursors into the host and allow normal host metabolism to synthesize compounds of interest. For example, using a mouse model with an intravenously-administered ^13^C/^15^N-labeled threonine as a tracer for newly-synthesized proteins, *Akkermansia muciniphila* and *Bacteroides acidifaciens* were identified as the most abundant *in vivo* utilizers of secreted host proteins using FISH combined with NanoSIMS [21]. Interestingly, this activity was partially abrogated in a gnotobiotic mouse model with a simplified community including *A. muciniphila* and *B. acidifaciens*, suggesting that their ecophysiology is dependent upon the background microbiota and highlighting the importance of studying microbial ecophysiology in natural, complex communities. Another strategy when isotopically-labeled compounds are not available is to perform incubations with the compound of interest in the presence of a general activity marker such as heavy water. By short *in vitro* incubation of mouse intestinal contents with the host glycoprotein mucin as a substrate and in the presence of heavy water, *A. muciniphila* and *B. acidifaciens*, as well as other members of the *Bacteroidetes*, were identified as utilizers of mucin by isotope quantification and single-cell sorting with Raman microspectroscopy followed by sequencing of sorted cells [38]. Kopf *et al.* used heavy water to estimate the growth rate of *Staphylococcus aureus* in cystic fibrosis sputum and to demonstrate substantial heterogeneity in growth rates using NanoSIMS imaging of single cells [57]. General activity has also been monitored *in vivo* using total ^15^N-labeled diets as all active microbes will incorporate N for growth. Recently, Oberbach *et al.* compared the active microbial community in colonic mucus of rats fed either a control diet or a high-fat diet with ^15^N using metaproteomics SIP and found that Verrucomicrobiaceae and Desulfovibrionaceae were enriched in the active fraction of the community in the high-fat diet [39].

These examples highlight how key organisms for specific metabolisms of interest can be identified and their metabolic products, as well as their contribution to metabolite pools, can be monitored without the need for prior knowledge of the genetic basis for these metabolisms. The identification of organisms mediating certain metabolisms is an important step for further investigation of these species using biochemical and, when possible, genetic techniques to elucidate the molecular basis of the metabolism. For example, recently ^13^C-labeled lysine was used in combination with ^13^C-NMR analysis and high-performance liquid chromatography to elucidate the lysine degradation pathway of *Intestinimonas* AF211, an abundant member of the human gut microbiota [58].

## Concluding Remarks and Future Directions

SIP technologies and methodologies have been rapidly developing, particularly in the area of single-cell analysis, which opens up new possibilities for studying host-associated microbiotas (see Outstanding Questions). A promising new area is the combination of Raman microspectroscopy with high-throughput sorting [59] to sort hundreds to thousands of labeled cells for molecular characterization or targeted cultivation. This higher throughput will allow a more complete description of the organisms as well as guilds of microbiome members involved in metabolisms of interest. Developments in mass spectrometry-based imaging, such as time-of-flight (TOF) SIMS, 3D OrbiSIMS [60], and MALDI-TOF MS, will also open the door for protein and metabolite SIP imaging in individual microbial cells. These technologies will assist in identifying proteins and metabolites produced by key organisms, which will help to resolve underlying metabolic pathways. An area that should receive further attention is the design of experiments that enable highly resolved quantitative physiological analysis *in situ* [57] and include mass balances and stable isotope-aided metabolic flux analysis among the cells in the microbiota and between the microbiota and host [14]. Combined analyses of host and microbiota physiology 61, 62, including simultaneous application of multiple tracers 16, 63, will help to unravel and quantify the multilayered fluxes of metabolites and elements between the microbiota and its host.

Outstanding Questions How can SIP be made more high-throughput for analysis of many samples?

Will secondary ion mass spectrometry (SIMS) technologies, such as 3D OrbiSIMS and TOF-SIMS, soon have the spatial resolution and sensitivity required for imaging of isotope-labeled metabolites in individual microbial cells?

How can SIP of host and microbiota physiology be better combined to obtain quantitative mass balance and flux data?
